# A Case for Microtubule Vulnerability in Amyotrophic Lateral Sclerosis: Altered Dynamics During Disease

**DOI:** 10.3389/fncel.2016.00204

**Published:** 2016-09-13

**Authors:** Jayden A. Clark, Elise J. Yeaman, Catherine A. Blizzard, Jyoti A. Chuckowree, Tracey C. Dickson

**Affiliations:** Menzies Institute for Medical Research, University of TasmaniaHobart, TAS, Australia

**Keywords:** amyotrophic lateral sclerosis, microtubules, dynamics, axon transport

## Abstract

Amyotrophic lateral sclerosis (ALS) is an aggressive multifactorial disease converging on a common pathology: the degeneration of motor neurons (MNs), their axons and neuromuscular synapses. This vulnerability and dysfunction of MNs highlights the dependency of these large cells on their intracellular machinery. Neuronal microtubules (MTs) are intracellular structures that facilitate a myriad of vital neuronal functions, including activity dependent axonal transport. In ALS, it is becoming increasingly apparent that MTs are likely to be a critical component of this disease. Not only are disruptions in this intracellular machinery present in the vast majority of seemingly sporadic cases, recent research has revealed that mutation to a microtubule protein, the tubulin isoform *TUBA4A*, is sufficient to cause a familial, albeit rare, form of disease. In both sporadic and familial disease, studies have provided evidence that microtubule mediated deficits in axonal transport are the tipping point for MN survivability. Axonal transport deficits would lead to abnormal mitochondrial recycling, decreased vesicle and mRNA transport and limited signaling of key survival factors from the neurons peripheral synapses, causing the characteristic peripheral “die back”. This disruption to microtubule dependant transport in ALS has been shown to result from alterations in the phenomenon of microtubule dynamic instability: the rapid growth and shrinkage of microtubule polymers. This is accomplished primarily due to aberrant alterations to microtubule associated proteins (MAPs) that regulate microtubule stability. Indeed, the current literature would argue that microtubule stability, particularly alterations in their dynamics, may be the initial driving force behind many familial and sporadic insults in ALS. Pharmacological stabilization of the microtubule network offers an attractive therapeutic strategy in ALS; indeed it has shown promise in many neurological disorders, ALS included. However, the pathophysiological involvement of MTs and their functions is still poorly understood in ALS. Future investigations will hopefully uncover further therapeutic targets that may aid in combating this awful disease.

## Introduction

Amyotrophic Lateral Sclerosis (ALS) is a late onset and ultimately fatal neurodegenerative disease characterized by the loss of upper and lower motor neurons (MNs) from the nervous system. Symptoms can be separated into bulbar (upper MN degeneration), or lumbar (lower MN degeneration) onset, describing the spread of pathology from the initial site. In addition to variance in the spread of pathology, ALS heterogeneity is further exacerbated by its representation as a spectrum disorder, with a range of clinical manifestations from cognitive dysfunction in frontotemporal dementia (FTD), pure motor phenotype in classical ALS, or a combination of both cognitive and motor dysfunctions (Ling et al., 2013). In ALS, cellular loss results in atrophy of cortical and spinal structures, and a loss of muscular innervation and associated muscle wastage (Mezzapesa et al., 2013; Turner and Swash, 2015). Neuronal cell loss is accompanied by reactive gliosis, and characteristic proteinacious intracellular inclusions (Ilieva et al., 2009; Peters et al., 2015).

Neurons are highly refined communicating cells that receive, process and relay information to their target cells, with MNs being particularly vulnerable to ALS-associated pathology. Factors contributing to the selective vulnerability of MNs in ALS include their large cell size and therefore energy dependency, their excitable nature coupled with a lack of buffering capacity, and their intimate relationship with neighboring non-neuronal cells. Many possible disease mechanisms have been proposed to account for the development and progression of ALS (reviewed in Peters et al., 2015). However, one such mechanism, the impairment of the axonal transport system, highlights the significance of the intracellular cytoskeleton, particularly the microtubules (MTs), in the neurodegenerative process.

## Microtubules Are Integral to Neuronal Function

Microtubules are structural cytoskeletal elements expressed in all eukaryotic cells. Their composition and general function are conserved between different cell types and organisms, and is essential for cell division and motility. MTs are of particular importance to neurons and are involved in a great number of additional functions including the development of neuronal cell polarity, the generation of neuronal compartments, growth cone mechanics, neurite remodeling and intracellular transport (Chen et al., 2006; Baas and Lin, 2011; Sakakibara et al., 2013). Within neurons, MTs form protofilaments from heterodimerized tubulin. These cylindrical structures are vitally important for the function of long extending axons, which have a high demand for intracellular transport of organelles, proteins and RNA granules. Therefore, it can be said MTs are essential to both the development and maintenance of the neuronal circuitry.

**Figure 1:**
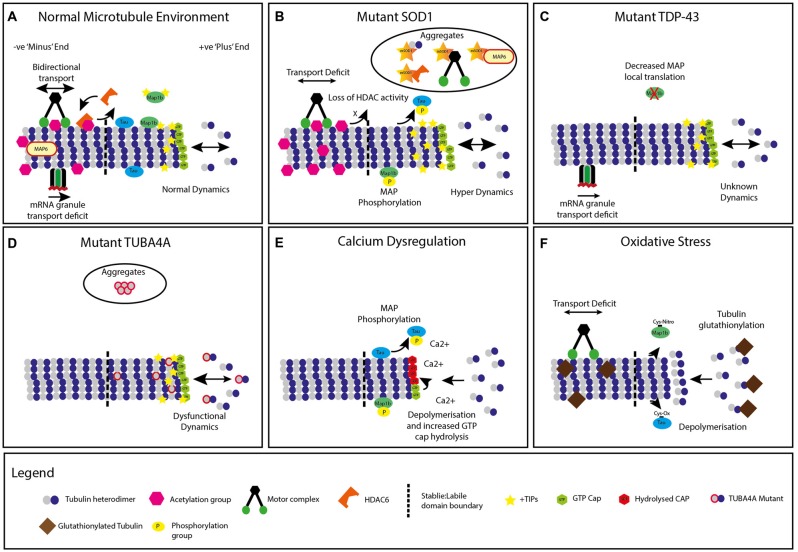
**Modifications to neuronal microtubules (MT) in amyotrophic lateral sclerosis (ALS). (A)** Normal microtubule +TIP dynamics and kinesin/dynein transport, mRNA granule transport and chemical modifications. **(B)** Mutant superoxide dismutase 1 (SOD1) expression leading to microtubule hyperdynamics, increased +TIP protein density, decreased transport, increased acetylation, phosphorylation of microtubule associated proteins (MAPs) and accumulation of microtubule protein containing aggregates. A global decrease in Histone Deacetylase (HDAC) activity is also present. **(C)** Mutant TDP-43 (TARDBP) expression causes dysfunction in mRNA granule transport. Decreased local translation of MAP mRNA is also implicated in TDP-43 mutants. **(D)** Mutant TUBA4A expression alters microtubule dynamics and network stability, with unknown impact on +TIP proteins, transport or chemical modifications. Select mutations are incorporated into intracellular aggregates. **(E)** Energy depletion and calcium dysregulation generates increased microtubule depolymerization, tubulin guanosine triphosphate (GTP) cap hydrolysis, and increased MAP phosphorylation. **(F)** Neuronal oxidative stress leads to tubulin glutathionylation, increased microtubule depolymerization, decreased axonal transport and alterations to MAPs, with unknown impact on classical chemical modifications or +TIP proteins.

MT protofilaments are comprised of dimers of α and β-tubulin, which through lateral interactions, form the characteristic MT structure (Desai and Mitchison, 1997). MTs undergo bouts of assembly and disassembly from their ends, a process termed dynamic instability (Mitchison and Kirschner, 1984; Figure 1A). A slow growing α-tubulin “minus end” and fast growing β-tubulin “plus end” (Allen and Borisy, 1974; Van Beuningen et al., 2015; Yau et al., 2016) can be accounted for by the guanosine triphosphate (GTP) cap model. GTP bound to β-tubulin confers plus end stability, and upon hydrolysis, leads to MT depolymerization (Desai and Mitchison, 1997; Nogales and Wang, 2006). This directionality in MT structure allows the formation of neuronal compartments and therefore confers neuronal polarity. The axonal compartment has exclusively distal facing positive polarity, or “plus end out” orientating MTs (Heidemann et al., 1981). Contrastingly, the somatodendritic compartment contains a mixed orientation of MT directions, with equal quantities of plus and minus end facing MTs (Yau et al., 2016).

Neurons, being relatively long-lived and stable cells, require a stable cytoskeleton. Neuronal MTs can be separated into two domains: labile and stable (Baas and Black, 1990; Baas, 2013). While mature neurons have an MT network consisting of both domains, the majority of the MTs are stable (Ferreira and Cáceres, 1989; Lim et al., 1989). These domains cannot be fully explained by the myriad of tubulin isoforms available in the genome (Tischfield and Engle, 2010). This functional diversity has instead been associated with domain-specific chemical modification to tubulin subunits, which aid in increasing the functionality of MTs, and in some cases, can impact upon their stability (Baas, 2013; Janke, 2014). These modifications to neuronal MTs include, but are not limited to, tubulin tyrosination, acetylation, polyamination, glutathyonilation, glutamylation and glycylation (reviewed previously in Janke and Kneussel, 2010; Janke, 2014). These post-translational modifications may confer stability to MT structure and alter binding affinity of MT-associated proteins, thereby altering their function. Although not being fully understood, these chemical modifications can also be utilized as molecular markers of MT network stability, and alterations to chemical modifications are thought to be attractive therapeutic targets for neurodegenerative disorders (d’Ydewalle et al., 2011; Taes et al., 2013).

Perturbations in MT and microtubule associated protein (MAP) functions have been implicated in a range of neurodegenerative diseases (Dubey et al., 2015), including Alzheimer’s disease (AD; Matsuyama and Jarvik, 1989), Parkinson’s disease (PD; Ren et al., 2003; Cartelli et al., 2010), Huntington’s disease (HD), various congenital developmental disorders (Tischfield and Engle, 2010), schizophrenia (Morris et al., 2003; Andrieux et al., 2006) and also ALS (Baird and Bennett, 2013; Smith et al., 2014). Of note the MT stabilizer, Tau (Drubin and Kirschner, 1986), is involved in the pathophysiology of AD (reviewed in Hanger et al., 2014); Mcmurray (2000); Stancu et al. (2014) as well as in other neurodegenerative diseases (collectively tauopathies) such as supranuclear palsy, corticobasal degeneration and Picks disease (reviewed in Cairns et al., 2004). Alterations to proteins involved in MT stability, dynamics and MT turnover also occur in PD (Alim et al., 2004; Yang et al., 2005; Gillardon, 2009). Similarly, MT involvement has also been established through 1-methyl-4-phenylpiridinium (MPP^+^; Cappelletti et al., 2005) and Rotenone-mediated PD (Ren et al., 2005) in *in vitro* models, with these found to destabilize the MT network. Furthermore, it has been highlighted that perturbations to MT dynamics also leads to MT-dependent transport impairment in a model of PD (Cartelli et al., 2010). The gene disrupted-in-schizophrenia-1 (DISC-1), whose mutations are associated with familial forms of schizophrenia, has been suggested to associate and interact with MT components (Morris et al., 2003; Callicott et al., 2005), further implicating MT alterations as a causal factor in multiple neurodegenerative disorders. Conversely, mutations to tubulin genes generally lead to disorders associated with dysregulated neurogenesis, due to abnormalities in neuronal migration, cellular division of progenitor cells, neuronal differentiation and induction of cell polarity. These disorders include lissencephaly, polymicrogyria, mirocephaly, cerebellar dysplasia and some occulomotor disorders (Francis et al., 2006; Guerrini et al., 2008; Tischfield et al., 2011).

In ALS, the consequence of MT dysfunction has classically been hypothesized as being due to the physical length of the axon in affected MNs, with alterations to MTs being thought to impact on axonal transport (De Vos et al., 2007; Millecamps and Julien, 2013). However, evidence for MT dysfunction having a primary role in ALS has significantly increased over the last 15 years. The recent identification of tubulin rare variants and their impact on MT function, specific interactions with mutant and pathological proteins as well as altered function of MT-associated proteins and signaling pathways which affect MT dynamics, have all been implicated in ALS pathogenesis. This review article will consider the role MTs play in disease pathogenesis and potential mechanisms that may have an impact on MT function in ALS.

## The Genetic Inheritance of ALS; Recent Insights into Microtubule Function

Familial genetics of ALS are being increasingly understood, in both the context of the mechanisms through which they act, as well as in the clinical phenotype presented (Marangi and Traynor, 2015; Peters et al., 2015; Turner and Swash, 2015). Moreover, the fact that MTs and their functions are a site of pathological convergence for various ALS mutations is becoming increasingly appreciated. The first identified causative mutation for ALS was in the gene coding for superoxide dismutase 1 (SOD1), which was also used to generate the first animal model of the disease, the widely used SOD1^G93A^ transgenic mouse (Rosen, 1993; Gurney et al., 1994). More than 160 different mutations to the gene encoding for SOD1 have been reported (Rosen, 1993; Al-Chalabi et al., 2012), accounting for approximately 20% of all familial forms of ALS (FALS; Andersen and Al-Chalabi, 2011; Robberecht and Philips, 2013). It has long been established that SOD1 mutations can cause axonal transport dysfunction, thus suggesting an interaction with the MT system (Warita et al., 1999; Ligon et al., 2005; Bilsland et al., 2010; Ikenaka et al., 2012). This has been reported to occur as early as embryonic day 13 in the SOD1^G93A^ mutant mouse (Kieran et al., 2005). Recently, evidence has been published supporting the hypothesis that alterations to MT dynamics are driving MN dysfunction in these mice (Fanara et al., 2007; Kleele et al., 2014; Figure 1B). Indeed, mutant SOD1 has been implicated in the alteration of signaling cascades that may affect proper MT functioning, however, this process is still poorly understood (Evans et al., 2000; Nguyen et al., 2001; Vadlamudi et al., 2005; Lopez-Fanarraga et al., 2007; Ikeda et al., 2011; Zyss et al., 2011; Bunton-Stasyshyn et al., 2015).

Similarly, discoveries of mutations in RNA-processing proteins such as TDP-43 (TARDBP) and fused in sarcoma (FUS) were both identified as a cause of FALS and FTD, and subsequently used to generate further models of ALS (Gitcho et al., 2008; Kabashi et al., 2008; Sreedharan et al., 2008; Kwiatkowski et al., 2009; Vance et al., 2009). TDP-43 is associated with transcription repression (Ou et al., 1995), the regulation of splice variants (Buratti and Baralle, 2001), mRNA stability and/or transport (Tollervey et al., 2011). In disease, both mutated and wild-type TDP-43 mislocalize from the nucleus to the cytoplasm and form cytoplasmic inclusions (Neumann et al., 2006). FUS is also a nuclear DNA/RNA binding protein and is involved in various aspects of gene expression through mRNA metabolism and mRNA transport. Mutated FUS, similar to TDP-43, is mislocalized to the cytoplasm due to a loss of nuclear import signaling (Dormann et al., 2010). The similar function and mislocalization of these two proteins, TDP-43 and FUS, indicates that familial insults in neuronal degeneration may have common mechanisms. It has been found that TDP-43 interacts with MTs in the MT-dependant transport of mRNP granules (Kanai et al., 2004; Alami et al., 2014; Figure 1C). This transport is particularly important due to TDP-43’s role in local mRNA translation of proteins in dendrites, axons and synapses, such as the neuromuscular junction (NMJ; Kanai et al., 2004; Belly et al., 2005; Fallini et al., 2012). Similarly, TDP-43 interacts directly with proteins involved in RNA transport (Freibaum et al., 2010). Mutated TDP-43 has recently been identified to impair axonal transport of mRNA *in vivo*, as well as the induced pluripotent stem cell (iPSC) derived MNs of patients with TDP-43 mutations (Alami et al., 2014). mRNP granule transport defects were independent of other transport reporter defects such as mitochondrial transport. It was hypothesized that diminished transport of mRNA to compartments such as the axon and NMJ may lead to a decrease in proteins particularly important for NMJ maintenance and survival (Polymenidou et al., 2011; Lagier-Tourenne et al., 2012).

In 2009, two independent studies identified the hexanucleotide repeat expansion of the non-coding region of reading frame 72 on chromosome 9 (C9ORF72); a mutation that occurs in both sporadic ALS (SALS) and FALS, also FTD, at greater frequency than other, previously known mutations (DeJesus-Hernandez et al., 2011; Renton et al., 2011). Initially it was postulated that this abnormal repeat could be transcribed and may have some toxic function, however the exact mechanism of pathogenesis remains unclear (DeJesus-Hernandez et al., 2011). More recently it has been proposed that the presence of the repeat expansion may result in “unconventional translation” (Ash et al., 2013; Todd and Petrucelli, 2016). This leads to five dipeptide repeat proteins, which accumulate in neurons (Mori et al., 2013). It is still unknown whether these repeat expansions lead to a gain or loss of function mechanism, however support for C9ORF72 gain of function is growing (Todd and Petrucelli, 2016). While function of this repeat expansion is still under investigation, Droppelmann et al. (2014) have highlighted that possible interaction with MTs may exist, due to the C9ORF72 homology to a guanine nucleotide exchange factor (GEF) that signals Rab-GTPases. Rab-GTPases are involved in membrane trafficking of proteins (Levine et al., 2013; Droppelmann et al., 2014) and hence, may be dependent upon MTs.

Although the majority of FALS cases follow a dominant pattern of inheritance, mutations to Alsin (ALS2) have been found to exhibit a recessive pattern of inheritance (Hadano et al., 2001). Mutations in the ALS2 gene have been associated with the development of juvenile onset ALS, as well as a range of other conditions such as primary lateral sclerosis and hereditary spastic paraplegia (HSP; Eymard-Pierre et al., 2002; Panzeri et al., 2006), with 12 different mutations identified to contribute to the development of such conditions (Chandran et al., 2007). Many of these mutations result in a premature stop codon, therefore rendering the ALS2 protein non-functional (Yamanaka et al., 2006). ALS2 is a GEF, which promotes guanosine diphosphate (GDP) release and GTP binding onto target proteins, as well as the stimulation certain signaling cascades (Tudor et al., 2005). ALS2 mutations have been suggested to contribute to the pathogenicity of ALS through the disruptions of Rab5-dependent exocytosis, endosome trafficking and also glutamate-associated excitotoxicity, which is considered a hallmark of ALS pathology (Devon et al., 2006; Hadano et al., 2006; Lai et al., 2006). Indeed, ALS2 acts through signaling cascades that impact MTs, and whose loss of function may generate MT dysfunction; a process that requires further research (Tudor et al., 2005).

Vascular endothelial growth factor (VEGF) is a polymorphic risk factor for ALS, and its expression is reduced in patients (Brockington et al., 2006, 2010). A mouse model that produces decreased levels of VEGF develops a motor neurodegenerative phenotype, with behavioral deficits and cellular loss (Oosthuyse et al., 2001). VEGF mice show decreased expression of the MAPs tau, MAP1b and MAP6, which has implications for stability of both the stable and labile domains of MTs in ALS (Brockington et al., 2010). Furthermore, genes relating to transport and dynein complex cargo loading are also down regulated. This occurs well before MN loss, but at the time of motor behavior phenotype generation. This may highlight a link between dysfunction of MTs and other identified genes, leading to MN dysfunction, followed by cellular demise (Brockington et al., 2010).

## Microtubule Protein Mutations: A Primary Cause of ALS

Recent research has highlighted that mutations to MTs can also initiate adult onset MN dysfunction, suggesting that MTs may be a primary driver for ALS pathophysiology (Al-Chalabi et al., 1999; Puls et al., 2003; Gros-Louis et al., 2004; Wu et al., 2012; Smith et al., 2014). Smith et al. (2014) found dominant negative mutated variants in the TUBA4A gene on chromosome 2 in FALS patients. These mutations were reported to cause classical spinal onset ALS, with upper and lower MN loss (Smith et al., 2014), and in some cases, FTD-like symptoms. The mutated region normally interacts with β-tubulin and the motor domain of kinesins and other MAPs (Liu et al., 2012; Howes et al., 2014). These variants were further shown to ineffectively form tubulin dimers and displayed a decreased incorporation into protofibrils, inhibiting MT network stability (Figure 1D). Similarly, other mutations affecting the conformation of tubulin proteins may subsequently alter the assembly of tubulin, resulting in an unstable MT structure (Tischfield et al., 2011).

TUBA4A is ubiquitously expressed in all cell types, but at high levels in the nervous system (Rustici et al., 2013; Smith et al., 2014). The expression of TUBA4A also increases over time, possibly illuminating why mutations in these genes cause later age disease phenotypes, unlike congenital tubulin mutations, which generate developmental disorders (Tischfield et al., 2011; Hersheson et al., 2013). Further supporting this, and as highlighted by Smith et al. (2014) expression of the β-tubulin subunit, TUBB4A, whose mutations cause adult onset disease Torsion Dystonia Type 4, increases over time, similar to the expression pattern of α-tubulin TUBA4A (Hersheson et al., 2013). Further impacts of tubulin alterations in ALS can be shown in SALS patients, where there is a down regulation of α-tubulin subunit genes (Jiang et al., 2005). However, how MT dynamics and transport are impacted as a result of TUBA4A mutations are still not clear. Future animal models of TUBA4A mutations will allow for the identification of its role in pathogenesis, particularly focusing on its age-dependant expression pattern.

Collectively, disease-causing mutations have shed light on an integral role for MTs in ALS. However, with the majority of ALS cases still seemingly sporadic, it remains unclear if altered MT function is the cause, or consequence, of upstream initiating pathogenic mechanisms.

## Pathogenic ALS Mechanisms Impact on Microtubule Structure and Function

Many mechanisms and molecular pathways involved in both the initiation and maintenance of ALS have been identified (Van Damme et al., 2005; Ferraiuolo et al., 2011; Peters et al., 2015). These include, but are not limited to, mitochondrial-dependant energy depletion, excitotoxicity and calcium dysregulation and cellular oxidative stress. The interplay between these disease mechanisms and insults to MTs are not well understood, however, it is becoming increasingly appreciated that MTs may act as a site for mechanistic convergence, as they are impacted by various pathogenic molecular mechanisms associated with ALS.

### Motor Neurons are Metabolically Demanding

One such pathological mechanism is mitochondria-dependant energy depletion. A study conducted by Park et al. (2013) highlighted that energy depletion itself could be a cause of MT depolymerization, which may in turn further promote energy depletion through the inability of the MTs to facilitate movement of mitochondria (Park et al., 2013; Figure 1E). Interestingly, MT pathology was identified prior to alterations to mitochondrial swellings, suggesting it may be a primary event in energy depletion and axon degeneration. This inability to recycle mitochondria may contribute to the abnormal accumulations of these organelles as observed in mutant SOD1 transgenic mice (Sotelo-Silveira et al., 2009). However, aberrant calcium dysregulation as a result of mitochondrial energy depletion, an identified toxic mechanism in ALS, does not seem to directly be a cause of MT dysfunction, suggesting that other, as yet unidentified, mechanisms may be at play (Park et al., 2013).

### Excitotoxicity and Calcium Dysregulation Affects Microtubules

Excitotoxicity has also been identified as a primary mechanism in the initiation and maintenance of ALS (Van Damme et al., 2005; Blizzard et al., 2015). Excessive influx and concentrations of intracellular Ca^2+^, whether from damaged mitochondria (Jaiswal, 2014), glutamatergic over stimulation, lack of glutamate clearance or a loss of Ca^2+^ buffering capacity, can lead to MN death (Heath and Shaw, 2002). Ca^2+^ concentrations also have an effect on MT-based transport of mitochondria through inhibition of attachment of kinesins to MTs (Wang and Schwarz, 2009). Indeed, excessive intracellular Ca^2+^ can lead to aberrant cyclin-dependent kinase 5 (CDK5) activity, which is particularly prominent in the SOD1 mouse model of ALS (Patzke and Tsai, 2002). Moreover, Ca^2+^ can interact directly with MTs, altering their dynamics (O’Brien et al., 1997; Figure 1E). Interestingly, heightened Ca^2+^ concentrations are sufficient to cause MAP containing MT preparations to depolymerize, due to an increase in β-tubulin GTP cap hydrolysis. Furthermore, Ca^2+^ can cause MAP2 loss and MT depolymerization in dendrites treated with NMDA, through calpain proteolysis of MAP2; however, it remains unclear if MT destabilization occurs prior to MAP2 loss (Hoskison et al., 2007). Excitotoxicity has also been shown to have an impact on retrograde transport machinery (Fujiwara and Morimoto, 2012). Indeed, this insult leads to caspase activation and subsequent cleavage and disintegration of the cytoskeleton, reported to be downstream of MT events (King et al., 2013). However, investigation of low dose, long lasting, chronic excitotoxic effects on both MT dynamics and transport are yet to be completed, as recognized ALS insults are chronic or accumulative in their nature. Investigations such as these will identify whether biologically relevant levels of neuronal excitotoxicity alter MT dynamics and transport first, or contrastingly activate the CDK5-p25 and caspase pathways, which lead to cellular pathology.

### Oxidative Stress and The Effects on Microtubules

Oxidative stress occurs due to the build-up of oxidative species, which while being a process of general aging of an organism, can cause uncontrollable oxidation of proteins or molecules, leading to cellular dysfunction. Therefore, homeostatic control of reactive oxygen species (ROS) is required for proper cell function. Uncontrollable ROS production and cellular oxidation leads to a number of neurodegenerative diseases (Andersen, 2004). Relevant to ALS, and in addition to Ca^2+^ toxicity, dysfunctional mitochondria can also drive uncontrollable ROS production (Tahara et al., 2009). While ROS are required for cytoskeletal remodeling and during axonal growth (Munnamalai and Suter, 2009; Wilson and González-Billault, 2015), aberrant ROS and an increased oxidative environment can lead to deleterious impacts on MTs, particularly oxidation of tubulin and selected MAPs (Landino et al., 2004). Although still poorly understood in neurons, increasing ROS in myocytes increase MT depolymerization (Drum et al., 2016). Both α- and β-tubulin contain Cys residues that have the capacity to oxidize (Landino et al., 2007; Wilson and González-Billault, 2015). Oxidative species added to purified tubulin preparations can cause a reduction in polymerization, and increase MT depolymerization, similar to that of increased Ca^2+^ levels (Landino et al., 2007; Figure 1F). Moreover, it has been established that oxidative stress affects MT integrity; this is evidenced by the presence of methionine sulphaoxides in the β-III tubulin in the brain of AD patients (Boutte et al., 2006). Furthermore, glutathionylation of tubulin, particularly in MNs, occurs during oxidative phases, altering MT dynamics and structure (Carletti et al., 2011). Indeed, the MAPs tau and MAP2 contain Cys residues, which when oxidize, impede MAP function, thus causing MT stability issues (Landino et al., 2004). Others have identified that recapitulating neuronal oxidative stress through the addition of hydrogen peroxide inhibits axonal transport, prior to mitochondrial dysfunction or axonal degeneration (Fang et al., 2012).

## Altered Regulation of Microtubule Dynamics has been Associated with ALS

Changes to microtubule dynamics in ALS have also been reported, particularly increases to MT dynamic instability. Fanara et al. (2007) identified that hyperdynamic MTs were present in the SOD1^G93A^ mouse model of ALS, and modulation of MT dynamics can ameliorate disease progression. A subsequent study found that MTs are indeed more dynamic, with an increase in end binding-protein 3 (EB3) +TIP comets on sciatic nerve axonal MTs in the SOD1^G93A^ mouse in comparison to unmutated individuals (Kleele et al., 2014). EB proteins are +TIP MAPs that bind to the growing phase of the labile domain of MTs, aiding in both dynamics and microtubule interactions with other intracellular objects. Increases in +TIP comets signify that the microtubule network is hyperdynamic. It was found that increased MT dynamics consequently slowed axonal transport, occurring presymptomatically (Fanara et al., 2007; Bilsland et al., 2010). Axonal transport dysfunction is then followed by an increase in neuronal pathology and subsequent mortality (Collard et al., 1995; Sasaki and Iwata, 1996; Williamson and Cleveland, 1999; Sasaki et al., 2004). Pharmacological amelioration of MT hyperdynamics not only reverses axonal transport deficits, but also improves clinical symptoms and survival (Fanara et al., 2007). This hints that hyperdynamics can indeed drive transport deficits, followed by cell demise; however, this process is still poorly understood. A possible mechanism through which SOD1 mice develop MT hyperdynamics may be through the interaction of mutant SOD1 and tubulin (Kabuta et al., 2009). Interestingly mutant SOD1/tubulin interactions do not diminish the free tubulin pool; however, it generates a destabilizing effect on MT.

Kabuta et al. (2009) suggest that homology exists between the site of mutant SOD1 binding to tubulin and the binding site of MT destabilizing agents such as colchicine and nocodazole. This may indicate that mutant SOD1 interacts in a similar manner to the MT destabilizing agents, increasing the dynamic instability of MT labile domains, leading to a reactive, hyperdynamic phenotype. A phenomenon less often considered in ALS research is the impact that intracellular aggregates, consisting of proteins such as misfolded SOD1 and neurofilaments (NFs), may have on microtubule dynamics. Indeed, these aberrant structures have the propensity to incorporate many different cytosolic proteins into the aggregate mass, with MT proteins being particularly prone in aggregate localization. This may be an interesting avenue of research as a recent study showed that decreasing the NFs in a *pmn* mice improves aberrant microtubule dynamics and instability that is associated with this model (Yadav et al., 2016).

An increasing and popular notion is that alterations to transport proteins, tubulin, other MAPs or the dysregulation of MT dynamics can result in aberrant MT structure and function, leading to either developmental disorders or degenerative phenotypes, such as that in ALS (Dubey et al., 2015). Mechanisms driving these alterations in MT dynamics are not well understood, nor are the hypothesized subsequent transport dysfunctions. Identification of whether dysfunction of MT dynamics is a common pathology between familial mutations and sporadic disease is paramount to understanding both disease onset and also its maintenance.

## Could Motor Protein Dysfunction Drive ALS Pathology?

Intracellular transport is a major function of neuronal MTs, with alterations in transport associated with a number of neurodegenerative diseases (reviewed in Millecamps and Julien, 2013). Cortical and spinal MNs are thought to be particularly vulnerable to transport dysfunction, due to their axonal length. It is becoming increasingly appreciated that alterations to MT dynamics precede, and thus may aberrantly affect, axonal transport, and that the resultant transport defect can have deleterious effects on neuronal function (Hurd and Saxton, 1996; Fanara et al., 2007; Bilsland et al., 2010; Cartelli et al., 2010; Dubey et al., 2015). The transport MAPs, kinesin and dynein, act as carriers for organelles, proteins and other cellular cargo in a directionally-dependant manner. Kinesin motor proteins transport cell cargo toward the plus end of the MT (anterograde), whereas dynein transport in the minus end direction (retrograde; Maday et al., 2014). Kinesin can have an impact on the stability of MTs, and expression of KIF5, a kinesin motor protein isoform, has been found to be decreased in the spinal cord and sciatic nerves of a mutant SOD1 mouse model (Maximino et al., 2014). This indicates that altered MT-dependent transport may be depleting MNs via the generation of an energy and signaling deficit. This is also substantiated in a study by Tateno et al. (2009), who showed that kinesin-associated protein 3 (KAP3), a kinesin subunit responsible for binding cargo such as choline acetyltransferase (ChAT), was selectively vulnerable to co-aggregation with misfolded SOD1 (Tateno et al., 2009). This phenomenon was also reported to occur in human SOD1 FALS patients, possibly illuminating a further source of MN vulnerability to dysfunctional transport of specific cargos in ALS.

Dynein, in conjunction with its molecular binding partner and activator, dynactin (DCTN1), is also vulnerable, both as a primary driver of ALS and also as a site of convergence of ALS insults (Ligon et al., 2005). Dynein interacts with mutant SOD1 and is located in proteinacious aggregates in SOD1 mice (Ligon et al., 2005; Figure 1B). Mutant SOD1 also interacts directly with the assembled dynein-dynactin complex, occurring prior to disease onset, at a similar age when retrograde dynein-mediated axonal transport dysfunction occurs in SOD1^G93A^ mice (Zhang et al., 2007; Bilsland et al., 2010). The functional consequence of this is yet to be determined, however detrimental impacts of transport dysfunction on the ubiquitin-proteasome system and protein autophagy may create a positive feedback loop, whereby proteins are caught in the incorrect cellular compartment, which compounds aggregation (Goldberg, 2003; Ström et al., 2008; Takalo et al., 2013). Similarly, bidirectional transport of mitochondria is affected presymptomatically, highlighting the cargo and directional specificity of axonal transport dysfunction in this model; however, the exact mechanism is not well understood (Bilsland et al., 2010). Mutations to the p150^Glued^ subunit of DCTN1 are associated with MN degeneration and ALS (Münch et al., 2004, 2005; Levy et al., 2006; Laird et al., 2008). The mutation distorts the folding of the MT-binding domain. An autosomal dominant variant has also been reported to concurrently cause FTD (Münch et al., 2005), highlighting the role of motor proteins in other neurodegenerative diseases.

Expressions of motor protein genes are altered in SALS patients and in mutant SOD1 mouse models. A reduction in dynactin-1 expression is observed, in the absence of alterations to kinesin and dynein expression; down regulation occurs prior to the deposition of NF protein aggregates (Jiang et al., 2007; Rustici et al., 2013). Furthermore, a polymorphism and reduced expression in kinesin-associated protein 3 (KIFAP3) correlates with an extended life span in SALS patients (Landers et al., 2009); however, how this affects transport is unknown. Interestingly, in mutant SOD1 mice, KIFAP3 expression in increased early in the diseases clinical course (Dupuis et al., 2000). Similarly, a number of cytoskeletal genes are altered in the SOD1^G93A^ mouse spinal cord and sciatic nerve, dependant on both age and MN sub compartment evaluated (Maximino et al., 2014). Similar gene expression changes were found in SALS patients, having a decrease in MAP2, MAP1b and tau protein expression, which is also seen in the mutant SOD1^G37R^ mouse model (Farah et al., 2003; Jiang et al., 2007). Motor proteins are also susceptible to alterations in tau levels, as observed in tauopathies, which retard anterograde motor transport (Ebneth et al., 1998). Tau alters the flux at which kinesin and dynein motor complexes bind to the MTs, but not the speed at which they travel the MT tracks (Trinczek et al., 1999), suggesting alterations to tau levels on MTs may impact transport where tau pathology and dysfunction is present. Indeed, multiple upstream effectors such as altered dynamics, MAP dysfunction and protein-protein interactions can produce aberrant axonal transport.

## Microtubule Associated Protein Alterations Also Contribute to ALS Pathology

MAPs facilitate MT functions such as cytoskeletal interactions, intracellular signaling and modification of MT dynamics and stability. Indeed, MAPs are thought to “tune” MT dynamics through both direct and indirect interactions (Tortosa et al., 2013; Sayas et al., 2015). Alterations to MAPs are observed in ALS, and are mainly due to the impact of dysregulated signaling and aberrant phosphorylation events. Indeed, MAP dysfunction is a downstream effect of many ALS-related pathological mechanisms. Aberrant hyper-phosphorylation and mutations to the MAP tau are associated with neurodegenerative disorders, such as AD, tauopathies and ALS/FTD (Hanger et al., 2014; Stancu et al., 2014; Huang et al., 2015; Baas et al., 2016). Tau is an axonal specific MAP that localizes to labile domains of MTs (Black et al., 1996). It is found throughout the axonal MT network. Tau alters MT dynamics by increasing the stability of the labile domain, preventing its depolymerization, while promoting assembly (Brandt and Lee, 1993; Panda et al., 1995). Tau influences MT dynamics, as it interacts directly with EB proteins and is required for the localization and density of EB proteins to the plus ends of MTs (Sayas et al., 2015). Another means by which tau mediates MT stability is its role in protecting MTs from the enzymatic severing by katanin (Kempf et al., 1996; Qiang et al., 2006), a process that is thought to be impacted upon in AD and tauopathies, due to the loss of tau-MT localization when tau is phosphorylated (Sudo and Baas, 2011). There are reports of hyper phosphorylated and insoluble tau deposits in cortical neurons of sporadic ALS patients (Strong et al., 2006). Furthermore, mouse models of mutant SOD1 show increased tau phosphorylation (Nguyen et al., 2001); and SOD1 *Drosophila* models show increased tau toxicity and neuronal degeneration attributed to by tau phosphorylation (Huang et al., 2015). Moreover, mouse models generating hyper activated CDK5, a kinase that causes aberrant tau phosphorylation, show increases in cytoskeletal disintegration and axonal swellings (Ahlijanian et al., 2000). Interestingly, lowering the expression of tau does not improve the phenotype of mutant SOD1 mice, suggesting that while it may play a role in disease progression, it is not necessary for neuronal pathogenesis (Taes et al., 2010). However, identification of tau-mediated alterations to microtubule dynamics in ALS are yet to be completed, and is a necessary avenue of research.

Another MAP that may play an important role in ALS is MAP1b. MAP1b has been shown to play a role in neurite and dendritic spine dynamics, having been identified to be enriched in zones of high MT dynamic instability, such as axonal growth cones and branch points and dendritic spines (Gonzalez-Billault et al., 2001; Tortosa et al., 2011; Villarroel-Campos and Gonzalez-Billault, 2014; Ketschek et al., 2015). Furthermore, MAP1b function can be regulated by phosphorylation at specific sites, which alters its interactions with other cytosolic proteins (Lucas et al., 1998; Villarroel-Campos and Gonzalez-Billault, 2014). MAP1b preferentially binds to, and stabilizes, the labile domain of MTs. MAP1b has recently been implicated in mutant TDP-43 pathology (Feiguin et al., 2009; Alami et al., 2014; Coyne et al., 2014). It was identified that mutated TDP-43 leads to a reduction in local NMJ translation of the MAP1b homolog *futsch* in a *Drosophila* model of ALS (Coyne et al., 2014). MAP1b also sequesters the plus TIP binding protein EB3 from the growing plus ends of MTs (Tortosa et al., 2013). MAP1b over expression leads to a loss of EB3 colocalization, and down regulation leads to an increase in EB3 binding to the MT, inducing MT stability defects and aberrant growth (Tortosa et al., 2013). This may not only be in part due to the physical interactions of MAP1b with EB3, but also due to MAP1b’s role in certain signaling cascades, in which end binding proteins and MT dynamics can be affected (Montenegro-Venegas et al., 2010; Villarroel-Campos and Gonzalez-Billault, 2014; Ketschek et al., 2015). Therefore, MAP1b, through multiple mechanisms, affects dynamic pools of MTs due to roles in signaling and by direct physical interactions (Tymanskyj et al., 2012).

Histone Deacetylase 6 (HDAC6) deacetylates lysine 40 on α-tubulin, which is the hallmark of MT stability. Alterations to the function and expression of HDAC6 therefore have implications for MT transport (Reed et al., 2006). Indeed, this is found to be the case in SOD1 mouse models of ALS. Interactions between HDAC6 and mutant SOD1 lead to the development of intracellular aggregates containing HDAC6 *in vitro* and consequently result in the inhibition of HDAC6 deacetylase activity (Gal et al., 2013). This sequestering of HDAC6 reduces its deacetylating action, resulting in greater tubulin acetylation. Furthermore, HDAC6 facilitates the degradation of poly-ubiquinated proteins, such as SOD1 and TDP-43, through the autophagosome (Kawaguchi et al., 2003; Lee et al., 2015). Permanent binding of HDAC6 to mutant SOD1 may account for the loss of deacetylase activity and the increase in SOD1 aggregates due to ineffective action of HDAC6 in protein degradation pathways, effectively providing a double-hit mechanism (Gal et al., 2013). Gal et al. (2013) hypothesized that the described SOD1-HDAC6 model leads to an increase in axonal transport in mutant SOD1 mice; therefore increasing the transport, spread and deposition of misfolded SOD1 aggregates. Deletion of HDAC6 *in vivo* delays disease progression in SOD1^G93A^ mice, highlighting a possible non-cell autonomous action of HDAC6 that may add to the diseases phenotype, or the gain of function of the SOD1-HDAC6 complex in these mice (Taes et al., 2013). However, the increase in acetylation in mutant SOD1 models is counter-intuitive to the identified increase in microtubule dynamics: acetylation is a marker of stable microtubule domains, highlighting the need for further research (Fanara et al., 2007; Gal et al., 2013; Kleele et al., 2014). Furthermore, HDAC6 has been identified as a substrate of TDP-43; TDP-43 and FUS act in a complex to regulate HDAC6 expression (Fiesel et al., 2010; Kim et al., 2010), indicating that a number of mutations may impact HDAC6 activity, although, these are yet to be explored.

Stable, polyaminated domains of neuronal MTs are associated with MAP6 (originally stable-tubule-only-peptide—STOP), a protein that confers cold stability on MTs. MAP6 also prevents destabilization of MTs by pharmacological means (Slaughter and Black, 2003; Baas et al., 2016). Microtubule dysfunction as a primary driver of neurological pathology has been highlighted by the development of the MAP6 knockout mouse model. Interestingly, MAP6 knockout mice develop a schizophrenic-like phenotype, which can be rescued by pharmacologically increasing MT stability (Andrieux et al., 2006). This is also associated with transport deficits, with evidence suggesting it may be driven by a loss of network stability; pharmacologically increasing MT stability ameliorates this phenotype (Daoust et al., 2014). Of further interest is the identified localization of MAP6 to NF spheroids in the cortex and spinal cord of ALS patients (Letournel et al., 2003). However, the exact impact this has on disease is still not understood. In addition, MAP6 has been found to impact dendritic lysosome transport and trafficking, which can be impaired by the expression of an FTD risk factor with TDP-43 pathology (Kim et al., 2010; Schwenk et al., 2014).

MTs also interact with other cytoskeletal networks, such as the neuronal microfilament actin, and the neuronal specific NFs and are known to influence MT structure and function. Although current understanding of the interactions between these filaments is not fully appreciated, shared signaling cascades (Wittmann and Waterman-Storer, 2005) and shared associated or connecting proteins (Goryunov and Liem, 2016) are potential mechanisms underlying this communication. Cytoskeletal elements may affect one another in a more direct mechanism. This is evidenced by key proteins associated with actin assembly, such as formins, which bind to and regulate MT dynamics (Bartolini et al., 2008). Alternately, the MT TIP protein EB1 also has binding sites on actin, but this binding is mutually exclusive, and therefore competitive exclusion affects the stability neuronal MTs (Alberico et al., 2016). Importantly, NF accumulations are present in several neurodegenerative diseases, including ALS, and reviewed previously (Vickers et al., 2009). It has been suggested that accumulation of NF contributes to axonal degeneration by impeding axonal transport, causing defects in the cells’ ability to maintain the synapse (Collard et al., 1995). Depletion of NFs has been observed to increase lifespan and improve the phenotype of SOD1 mice (Williamson et al., 1998). While aggregation of misfolded proteins such as SOD1 and NF in ALS has been suggested to cause axonal transport dysfunction, the sequestration of toxic, misfolded protein or hyper phosphorylated proteins into subcellular compartments may in turn be neuroprotective (as previously reviewed in Patzke and Tsai, 2002; Takalo et al., 2013). This sequestration potentially reduces toxic oligomer interaction with endogenous proteins required for cellular function, or may act as a “sink” for aberrant phosphorylation; and therefore may extend the life of the cell. Alternately, this compartmentalization may detrimentally cause the sequestration of endogenous proteins that preferentially bind to aggregate proteins, and is still a cause for debate (Tateno et al., 2009).

Collectively, accumulating evidence indicates that MTs and their associated proteins may play an important role in both the initiation and progression of ALS. MT dysfunction may sit on a pathological continuum, whereby MTs can act as the primary driver of MN degeneration, or where other genetic or molecular mechanisms converge to cause MT pathology. Indeed, MT dysfunction in ALS points to aberrant alterations in dynamics, with resultant dysregulated axonal transport driving disease pathogenesis and ultimately cytoskeletal and cellular destruction. Thus these disease processes offer attractive targets for therapeutic intervention.

## Targeting Neuronal Microtubules for Therapy: An Approach for ALS

The only available treatment for ALS is the anti-excitotoxic drug, Riluzole. Riluzole acts on the presynaptic neuron to limit the release of glutamate into the synapse therefore reducing the excitotoxic effect of glutamate on the postsynaptic cell (Bensimon et al., 1994). Notwithstanding the reduction in excitotoxicity, treatment strategies involving riluzole have limited effectiveness, and are only able to extend patient life by approximately 3–6 months (Gurney et al., 1998). ALS is a multi-factorial disease, with many cellular components affected; therefore there are a range of available targets for therapy, however many of them have had limited success (Turner and Talbot, 2008). This review has discussed MT involvement in ALS and in particular how dynamics and function are impaired in disease states. Pharmacological manipulation of MTs to improve disease phenotype offers an attractive target in ALS. Indeed, this therapeutic approach has previously been undertaken in multiple neurodegenerative disease models, including ALS.

Fanara et al. (2007), established that hyperdynamic neuronal MTs were present in a SOD1 mouse model of ALS and administered the MT modulating agent noscapine to attenuate this phenotype. Noscapine, which can cross the blood brain barrier (BBB) effectively dampens hyperdynamics, leading to less depolymerization and polymerization events from occurring at the growing plus tip of the MT (Landen et al., 2002, 2004). Noscapine treatment extended lifespan, attenuated MT dynamics and normalized aspects of axonal transport. This in itself gives evidence for hyperdynamics driving transport deficits in these mice. However, more cargo-specific and rate-specific transport assays are required to identify if this is the case. Moreover, this study provides supporting evidence for the use of MT stabilizing agents in the treatment of ALS.

HDAC6 inhibitors and HDAC knockout mice have also been trialed to improve outcomes in ALS models, with the intention of increasing the acetylation of stable MTs to improve stability and axonal transport. The inhibition or removal of HDAC6 was found to improve the phenotype of mutant SOD1 mice (Taes et al., 2013). However, axonal transport was not directly measured. A similar study utilizing HDAC6 inhibition in Charcot-Marie-Tooth (CMT) disease showed an improvement in axonal mitochondrial transport, supporting HDAC6 inhibition as a candidate therapeutic for multiple neurodegenerative diseases (d’Ydewalle et al., 2011).

Direct MT stabilizing agents have been previously used in the medical setting in the treatment of cancers, as the addition of these agents perturb the formation of the mitotic spindle and therefore inhibit cell division (Schiff et al., 1979). The most studied MT targeting agent taxol has, in the last decade, developed a newfound use in modulating MTs in neurodegenerative diseases (Michaelis et al., 2006; Brunden et al., 2011; Das and Miller, 2012; King et al., 2013). At high doses, taxol treated systems develop over-stabilized MTs, preventing cell division in cancer cells; however, this can generate a painful peripheral neuropathy (Reyes-Gibby et al., 2009). At lower doses taxol has been found to limit MT depolymerization and stabilize the MT network in a number of neurodegenerative models (Michaelis et al., 2006; Brunden et al., 2011; Das and Miller, 2012; King et al., 2013). Indeed, treatment of an *in vivo* tauopathy model with taxol improves MN fast axonal transport, highlighting the therapeutic potential of taxol for disorders effecting MNs (Zhang et al., 2005). The range of taxol concentrations which yield beneficial effects on neurodegeneration are very narrow (Shemesh and Spira, 2011). Coupled with the off target effects identified due to taxol therapy, limited crossover at the BBB and the dramatic alterations to MT stability that it delivers, the benefits of taxol administration in neurodegenerative disease have been brought into question (Baas and Ahmad, 2013; Baas, 2014). In relation to ALS, taxol has been shown to reduce MT disintegration in an *in vitro* kainic acid excitotoxic model of ALS (King et al., 2013). Excitotoxicity exposure was reported to induce MT instability upstream of caspase-3 activation, which is mitigated with taxol treatment. However, as characterization of MT dynamics has not been completed with regard to excitotoxicity due to kainic acid exposure, it is difficult to assess whether taxol is indeed limiting the hypothesized dynamic instability of MTs, or preventing the breakdown of the MT network. Indeed, inhibition of caspase-3 activation in other excitotoxicity models reports neuroprotection and increased cell survival (Chen et al., 2001). However, it is important to note that Taxol derivatives, which exhibit higher BBB permeability, need to be investigated to determine if this form of MT stabilization is a viable treatment for ALS.

## Conclusion

MTs play a fundamental role in normal neuronal functions. MT dysfunction has been implicated in the pathogenesis of a number of neurodegenerative diseases including ALS, however, the precise mechanisms underlying this dysfunction are not fully understood. Currently our understanding of MT involvement in ALS suggests that generation of hyperdynamic MTs, transport dysfunction and alterations to cytoskeletal gene and protein expression may help drive disease pathogenesis. Identification of the chain of events, which lead to the dysfunction of the MT network in ALS, is paramount to our understanding of how MTs are involved in disease initiation and maintenance. Indeed, increasing evidence suggests that MT dysfunction is both a primary driver of pathology, and also a site for pathological convergence from associated familial and molecular ALS mechanisms. Modulation of these events and MT structural integrity is an attractive therapeutic target, with benefits to this approach being shown in other models of disease. Increasing our knowledge of the mechanisms behind MT dysfunction in ALS will hopefully uncover many more targets to manipulate pharmacologically to extend life or cure this debilitating and ultimately fatal disease.

## Author Contributions

JaAC (primary author) drafted the majority of the manuscript, along with the initial planning. EJY contributed to the remaining text of the manuscript, initial planning and through literature research of key themes. Various disease mechanisms were investigated by CAB, who also had a primary role in the planning and revision of the manuscript. JyAC has experience with the literature surrounding therapeutic treatment, and also played a primary role in the planning and revision of the manuscript. TCD contributed to the initial planning, literature research and revision of the manuscript. JaAC, EJY, CAB, JyAC and TCD all agreed for the final version to be published and agree to be accountable for all aspects of the work.

## Funding

This project was funded through the generous support provided by the National Health and Medical Research Council, the Motor Neurone Disease Research Institute of Australia, the Masonic Centenary Medical Research Foundation of Tasmania, the Dwyer Family, the Cape Hope Foundation and Diagnostic Services Pty Ltd.

## Conflict of Interest Statement

The authors declare that the research was conducted in the absence of any commercial or financial relationships that could be construed as a potential conflict of interest.
